# Genotyping of Single Nucleotide Polymorphisms Using Allele-Specific qPCR Producing Amplicons of Small Sizes Directly from Crude Serum Isolated from Capillary Blood by a Hand-Powered Paper Centrifuge

**DOI:** 10.3390/diagnostics9010009

**Published:** 2019-01-11

**Authors:** Gustavo Barcelos Barra, Ticiane Henriques Santa Rita, Daniella Paniago Jardim, Pedro Góes Mesquita, Camila Santos Nobre, Rafael Henriques Jácomo, Lídia Freire Abdalla Nery

**Affiliations:** 1 Sabin Laboratory, Brasília 70632-300, Brazil; ticihenriques@gmail.com (T.H.S.R.); daniellapaniago@gmail.com (D.P.J.); pedrogm@gmail.com (P.G.M.); camila.nobre@gmail.com (C.S.N.); rhjacomo@gmail.com (R.H.J.); lidia@sabin.com.br (L.F.A.N.); 2 Post-Graduation of Health Science, University of Brasília, Brasilia 70910-900, Brazil

**Keywords:** single nucleotide polymorphism, real-time polymerase chain reaction, serum

## Abstract

The cell-free genomic DNA (gDNA) concentration in serum ranges from 1500 to 7500 copies/mL within 2 h after phlebotomy (6–24 times the concentration observed in plasma). Here, we aimed to evaluate the gDNA size distribution in serum with time after coagulation and to test if crude serum can be directly used as a source of gDNA for qPCR. Next, we investigated if single nucleotide polymorphisms (SNPs) could be genotyped directly from the crude serum isolated from capillary blood using a hand-powered paper centrifuge. All tested PCR targets (65, 100, 202 and 688 base pairs) could be successfully amplified from DNA extracted from serum, irrespective of their amplicon size. The observed qPCR quantitation cycles suggested that the genomic DNA yield increased in serum with incubation at room temperature. Additionally, only 65 and 101 base pair qPCR targets could be amplified from crude serum soon after the coagulation. Incubation for 4 days at room temperature was necessary for the amplification of PCR targets of 202 base pairs. The 688 base pair qPCR target could not be amplified from serum directly. Lastly, serum was successfully separated from capillary blood using the proposed paper centrifuge and the genotypes were assigned by testing the crude serum using allele-specific qPCR, producing small amplicon sizes in complete agreement with the genotypes assigned by testing the DNA extracted from whole blood. The serum can be used directly as the template in qPCR for SNP genotyping, especially if small amplicon sizes are applied. This shortcut in the SNP genotyping process could further molecular point-of-care diagnostics due to elimination of the DNA extraction step.

## 1. Introduction

The cell-free genomic DNA (gDNA) concentration in serum ranges from 1500 to 7500 copies/mL within 2 h after phlebotomy (6–24 times the concentration observed in plasma) because the blood clotting process disrupts some leukocytes releasing their content into the liquid phase of this specimen. Furthermore, the gDNA concentration in serum increases with storage of the collection tube at 4 °C, indicating that there is a continuous release of gDNA from the blood clot to serum [1]. This high gDNA yield found in serum would allow its direct use for genotyping of clinically relevant single nucleotide polymorphisms (SNPs), exempting the requirement for DNA extraction. Moreover, the continuous release of gDNA from the blood clot to serum could be explored for gDNA enrichment in this specimen ex vivo, such an approach has recently been applied for rapid turnaround red cell genotyping [2].

Protocols using serum directly in quantitative PCR (qPCR) for detection of infectious agents have been published [3,4,5,6,7,8], indicating that serum is compatible with direct use in qPCR. Regarding the genotyping of SNPs directly from serum, Ulvik and colleagues described a protocol with that purpose in 2001 [9], however, the specimen needed to be dried before the qPCR to avoid the complete inhibition of the reaction. In fact, the direct use of crude serum for SNP genotyping would be more feasible because the qPCR master mixes were added by components and/or modified enzymes that diminish the impact of inhibitors over the reaction [6,7,10,11].

Regarding the advantages, the exemption of DNA extraction would further the molecular point-of-care diagnostics—such as quick genotyping assays, useful for personalized therapies [12,13]—especially if the tested specimen could be easily collected and processed. A hand-powered ultra-low-cost paper centrifuge used for serum separation from blood clot was recently described [14] and could be used for the above-cited purpose. On the other hand, it is important to highlight that blood DNases are active in serum [15]. Consequently, fragmented genomic DNA could be more prevalent than high molecular weight DNA in these specimens, meaning that higher sensitivity could be achieved by designing primers that amplify PCR products of small sizes.

Thus, the aims of this study were to evaluate: (a) The cell-free gDNA size distribution in serum along one week of storage at room temperature; (b) if crude serum (without any treatment) can be directly used as a source of gDNA for qPCR, exempting the requirement for DNA extraction; (c) if clinically relevant SNPs can be genotyped directly from crude serum isolated from capillary blood using a hand-powered paper centrifuge.

## 2. Methods

### 2.1. Ethics

The Hospital Oftalmológico de Brasilia-HOB research ethical committee approved this study (registry CAAE 50111515.0.0000.5667, approval date: 15 September 2015) and written informed consent was obtained from each participant.

### 2.2. Volunteers

The study enrolled 69 volunteers: 15 for the experiments evaluating the cell-free gDNA size distribution in serum and 54 for the experiments regarding the SNP genotyping.

### 2.3. Cell-Free gDNA Size Distribution in Serum along One Week of Storage at Room Temperature and Amplification of Genomic DNA Directly from Crude Serum Exempting the DNA Extraction

Venous blood was drawn by venipuncture in five Vacuette Z Serum Clot Activator 4 mL tubes (Greiner-bio-one, Kremsmunter, Austria). Each tube was stored for 0, 1, 2, 4 or 7 days at room temperature before serum separation (2000× *g* for 10 min). Serum (2 μL) was directly used in the qPCR reactions or submitted for DNA extraction (900 μL) using Nuclisens Easymag System (Biomérieux, Marcy-l'Étoile, France)—according to generic protocol 2.1.1 with the addition of 50 μL of the magnetic silica particle suspension and elution in 110 μL. The extracted DNA (5 μL) and crude serum (2 μL) were submitted to four distinct qPCR reactions, resulting in amplicons of different fragment sizes (65, 100, 202 and 688 bp). The qPCR reactions consisted of 10 μL of Maxima probe/ROX qPCR master mix (Thermo Scientific, Waltham, MA, USA), 1 μM of each primer, 0.5 μM of probe (all from IDT DNA Technologies, Coralville, IA, USA) (described in Table 1), 5 μL of extracted serum DNA or 2 μL crude serum in a total volume of 20 μL (supplemented with nuclease-free water). The thermal cycling was carried out in a StepOne™ Real-Time PCR System (Applied Biosystems, Foster City, CA, USA) with the following run conditions: Denaturation for 10 min at 95 °C; followed by 45 cycles of 15 s at 95 °C and 1 min at 60 °C with a single fluorescence measurement at the end of the extension.

### 2.4. SNP Genotyping Directly from Crude Serum Isolated from Capillary Blood Using a Hand-Powered Paper Centrifuge

Capillary blood (~100 μL) was drawn by finger prick and collected by dripping into two different microcentrifuge tubes (1.5 mL and 200 μL). The 1.5 mL tube contained 1 mL water to induce hemolysis and the remaining white blood cells were submitted to the Chelex-100 DNA extraction method [16].

Next, we adapted the paper centrifuge (paperfuge) described by Bhamla and colleagues [14] for 200 μL microcentrifuge tubes. This centrifuge was inspired by the toy known as whirligig (or buzzer). The adapted paperfuge is composed of a paper disc (12 cm in diameter) made of laminated craft paper (grammage of 240 g/m^2^) with two buttons of four holes (20 mm in diameter) attached in the center, one per side of the disc. A polyethylene braided fishing line of 0.48 mm was passed through each hole one time, leaving 15–20 cm of thread on each side of the paper disc. Two wood pencils were attached at each end of the thread to allow the manual spinning. Four equidistant transversal cuts (relative to the perpendicular diameter of the circle) of 1 cm were done 1 cm from the border, in order to hold the tubes. Thus, four tubes could be centrifuged at the same time. The tubes containing ~100 μL of coagulated blood were placed in the transversal cuts and the paper centrifuge was spun for four minutes in order to separate the serum. During the centrifugation itself, the thread and the paper disk stayed in the vertical and horizontal positions, respectively. The force is applied by pulling one pencil up and the other down with each hand. Blood was collected 4 h before the serum separation and 2 μL of the serum were used directly in the qPCR. A video showing the centrifuge construction can be found in [17].

The SNPs were genotyped using the Amplification Refractory Mutation System (ARMS) [18]—also named allele-specific PCR—adapted for qPCR. The allele-specific qPCR reactions consisted of 10 μL of 2× Maxima SYBR green/ROX qPCR master mix (Thermo Scientific), 1 μM of the common primer and 1 μM of an allele-specific primer (IDT DNA Technologies) (described in Table 2), 5 μL of extracted DNA or 2 μL crude serum in a total volume of 20 μL (supplemented with nuclease-free water). Two parallel reactions, one specific for each allele of the SNP, were necessary for the genotyping. The thermal cycling was carried out in a StepOne™ Real-Time PCR System (Thermo Fisher, Waltham, MA, USA), with the following conditions: Denaturation for 10 min at 95 °C; followed by 50 cycles of 15 s at 95 °C and 1 min at 60 °C (for gDNA) or 1 min at 50 °C (serum DNA) with a single fluorescence measurement at the end of the extension. The amplicon specificities were analyzed using melting curves, which consisted of 15 s at 95 °C, 1 min at 60 °C and a gradual increasing of the temperature from 60 °C to 95 °C in 0.3 °C steps with fluorescence reading after each 0.3 °C step. With an excess of PCR cycles, mismatches at the primer 3’-end usually do not refract the amplification; instead, they just delayed amplification of the specific target [19], so both allele-specific reactions amplified the same genomic DNA. As qPCR is kinetic, it allowed the visualization of the fluorescence increasing over time, thus one reaction worked as an internal control for the other and the genotypes were assigned considering the reaction with lowest quantitation cycle (Cq). If similar Cqs were observed in both reactions, it indicated a heterozygote genotype (see Figures 3–6).

### 2.5. Statistical Analysis

The degree of agreement between the test methods (serum) and the comparative methods (extracted DNA) was quantified using Kappa statistics with three categories [20]. A significant departure of genotype frequency from the Hardy–Weinberg equilibrium for each SNP was estimated [21].

## 3. Results

### 3.1. Cell-Free gDNA Size Distribution in Serum along One Week of Storage at Room Temperature and Amplification of Genomic DNA Directly from Crude Serum Exempting the DNA Extraction

**Figure 1:**
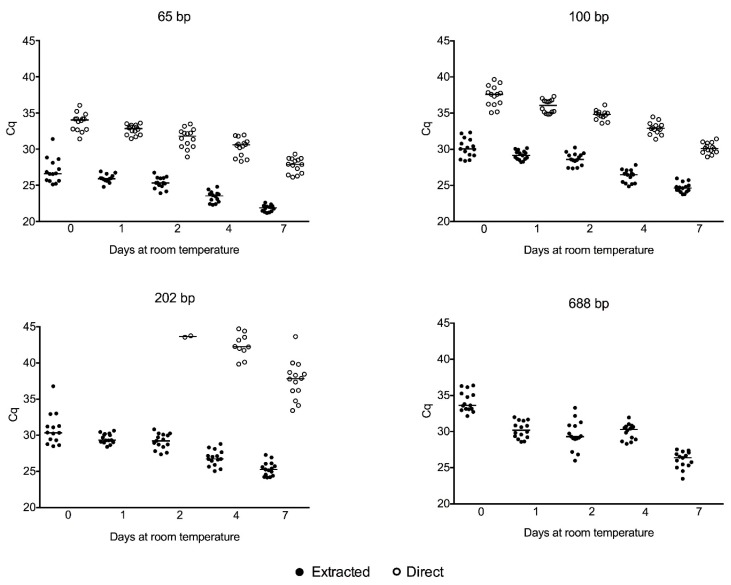
Quantitation cycle (Cq) values for amplicons of different sizes using DNA extracted from serum (closed circles) and crude serum directly (open circles) as template for qPCR. The line represents the median value.

Using the DNA extracted from serum, the tested PCR targets could be successfully amplified, irrespective of their amplicon sizes (65 base pairs (bp) (*RNase P* gene), 101 bp (*JAK2* gene), 202 bp (*SBP2* gene), and 688 bp (*ALMS* gene)). For all of them, the quantitation cycle (Cq) values showed a stepwise decline over time (Figure 1) suggesting that genomic DNA increases in serum with incubation at room temperature. Additionally, the Cq values tended to be lesser for the reaction amplifying small amplicons (65 bp) compared to reactions amplifying larger amplicons (101, 202 and 688 bp) suggesting higher yields of small DNA fragments in serum.

Moreover, the 65 and 101 pb PCR targets could be amplified using the crude serum (exempting the requirement for DNA extraction) from day 0 to day 7, in 15 out of 15 volunteers. The 202 bp PCR target could be detected in 2 out of 15 volunteers on day 2, in 10 out of 15 volunteers on day 4 and in 14 out of 15 volunteers on day 7. The 688 bp PCR target could not be detected at any time point until 7 days after blood collection (Figure 1). These results suggest that PCR targets with small amplicon sizes (<101 bp) can be amplified directly from crude serum soon after the coagulation, and incubation for at least 4 days at room temperature is necessary for the amplification of PCR targets with fragment size higher than 101 bp.

### 3.2. SNP Genotyping Directly from Crude Capillary Blood Serum Isolated Using a Hand-Powered Paper Centrifuge

Four genetic variants were selected to be genotyped directly from the crude serum (Table 3). Their minor allele frequencies (MAF) were higher than 0.25, to find all possible genotypes with a small number of samples.

**Figure 2:**
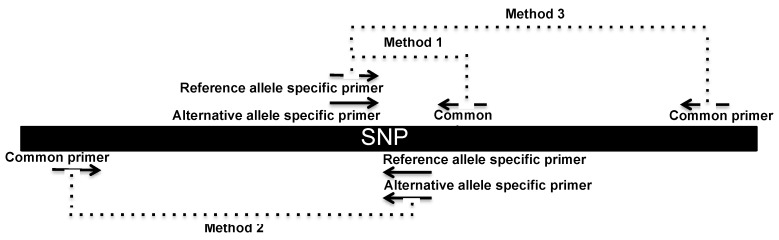
Allele-specific qPCR priming strategies used in methods 1, 2 and 3 for the genotyping of rs1801131, rs4939827, rs4779584 and rs3802842. Note: primer details can be found in Table 2.

In these experiments, an amplification primer set producing amplicons of small size was applied (method 1). The results observed using method 1 were compared to the results of the other two distinct methods, which were composed of primer sets that produce amplicons of conventional sizes (methods 2 and 3). Methods 2 and 3 were applied to genomic DNA extracted from blood. The amplification strategy of all three methods can be found in Figure 2.

**Figure 3:**
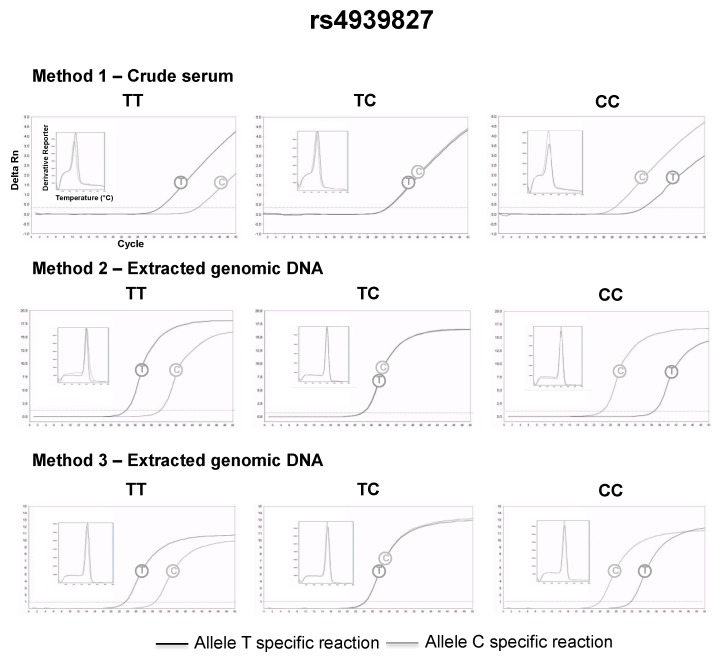
Representative rs439827 genotyping using method 1 (2 μL of crude serum separated 4 h after the capillary blood collection), 2 and 3.

**Figure 4:**
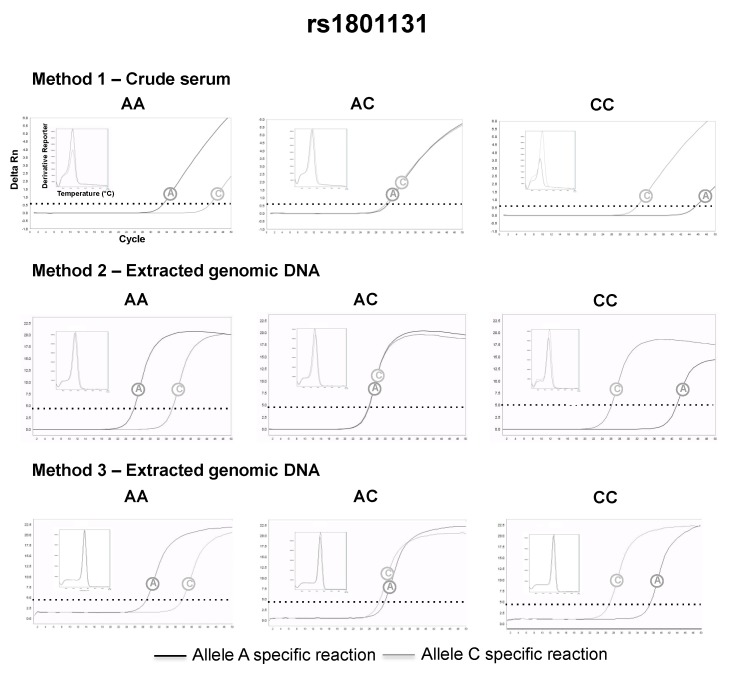
Representative rs1801131 genotyping using method 1 (2 μL of crude serum separated 4 h after the capillary blood collection), 2 and 3.

**Figure 5:**
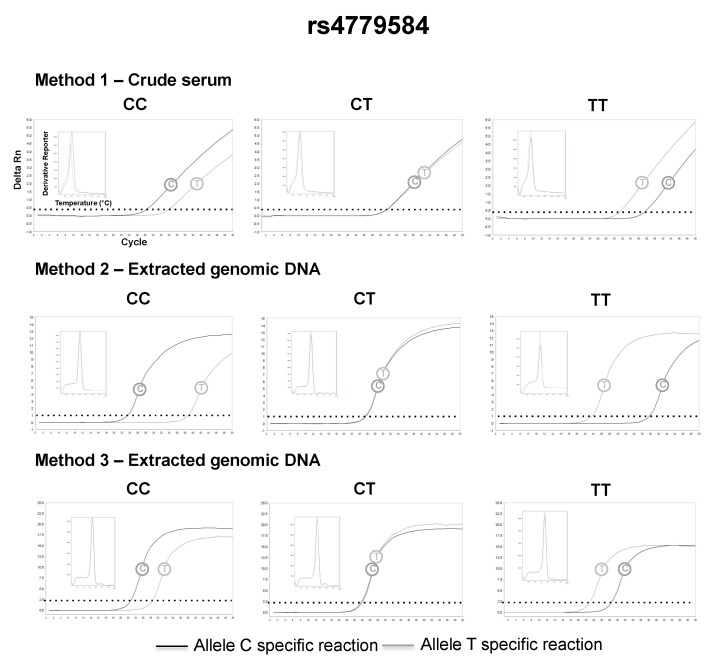
Representative rs4779584 genotyping using method 1 (2 μL of crude serum separated 4 h after the capillary blood collection), 2 and 3.

**Figure 6:**
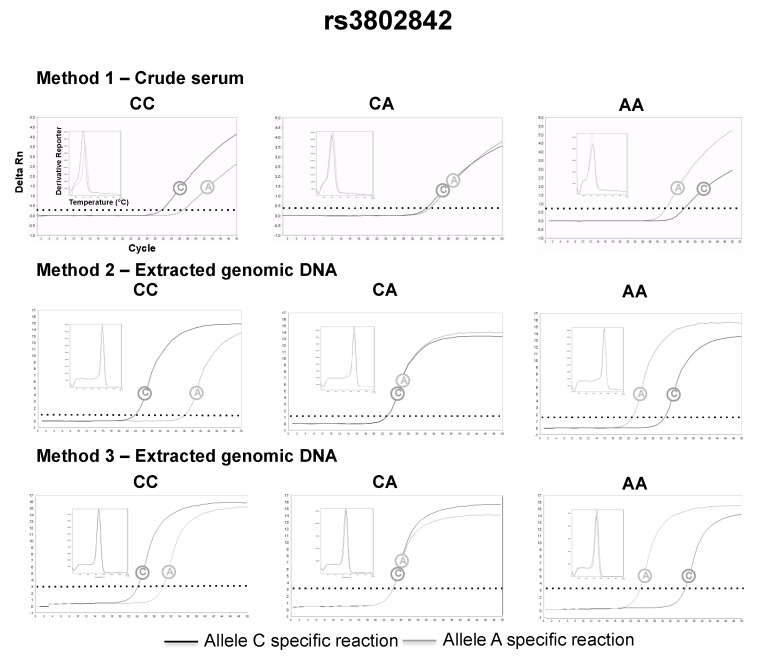
Representative rs3802842 genotyping using method 1 (2 μL of crude serum separated 4 h after the capillary blood collection), 2 and 3.

The serum was successfully separated from capillary blood using the proposed paper centrifuge, and the following genotypes could be retrieved directly from volunteers’ crude serum, using allele-specific qPCR and the small size amplicon strategy (method 1): 26 (48%) AA, 19 (35%) AC and 9 (17%) CC for rs1801131; 15 (28%) TT, 20 (37%) CT and 19 (35%) CC for rs4939827; 28 (52%) CC, 22 (41%) CT, 4 (7%) TT for rs4779584; 31 (57%) AA, 21 (39%) AC and 2 (4%) CC for rs3802842. The genotypes assigned by method 1 were in complete agreement with the genotypes assigned by method 2 and method 3 (Kappa = 1, a perfect agreement at all instances). Representative qPCR genotyping using crude serum and their comparison with DNA extracted from whole blood can be found in Figure 3, Figure 4, Figure 5 and Figure 6. All genotype distributions were in Hardy–Weinberg equilibrium (*p* >0.05% for all).

## 4. Discussion

We evaluated the cell-free gDNA size distribution in serum along one week of storage at room temperature. The observed results demonstrated that PCR products of different sizes could be amplified using genomic DNA extracted from serum as the template. The tested amplicon sizes were sufficient for the majority of genomics applications. Additionally, there was a direct correlation between the amplicon size and the Cq values, suggesting that small DNA fragments (<100 bp) are more prevalent in serum compared to DNA of higher fragment sizes (Figure 1). However, we cannot exclude if the smaller Cq values for smaller amplicons are secondary to the better amplification efficiencies of smaller targets.

Moreover, the amount of genomic DNA could be enriched in the serum if the specimen remains in contact with the blood clot at room temperature (the blood clot was the unique available source of DNA in the tube). So, the molecular diagnosis community can explore this phenomenon to increase the gDNA levels in the primary serum sample (e.g., performing sample transportation at room temperature). Serum can be an alternative to whole blood for molecular assays. It is friendlier for the majority of DNA extraction methods compared to whole blood because it has less cellular and protein content and less known PCR inhibitors (e.g., EDTA and hemoglobin [22]).

Our results also demonstrated that crude serum could be used directly as template in qPCR, exempting the requirement for DNA extraction. Generally, the DNA extraction is the most laborious step in the SNP genotyping process. Thus, the use of crude serum as a template makes these tests more practical. Patient samples are transferred to multiple tubes during the DNA extraction. The use of crude serum directly from the primary collection tube would enhance the quality assurance of genotyping assays, due to the lesser chance of pipetting mistakes.

Besides these advantages, the amplification of genomic DNA directly from serum is restricted by the amplicon size; only PCR products with less than 101 bp could be consistently amplified from crude serum soon after the coagulation. For consistent amplification of larger amplicons, incubation of four days at room temperature was necessary. In some situations, this period could make the assay turnaround time impractical. Small amplicon sizes are recommended for qPCR direct from serum. Furthermore, these results reinforce the observation that DNA fragments with less than 100 bp are more prevalent in serum compared to DNA fragments of greater sizes. This is likely because DNA in serum is cleaved to the mononucleosomal DNA fragment sizes by blood DNases [23], which are active in this specimen [15]. Additionally, apoptosis of white blood cells in the blood clot is the possible mechanism responsible for the DNA enrichment in serum. Apoptosis releases degraded DNA [24].

Next, we reduced the amplicon sizes (~50 bp) of some allele-specific primer sets to test if clinically relevant mutations could be genotyped directly from the crude serum (crude serum separated 4 h after the capillary blood collection). The results observed using amplicons of small sizes in crude serum were in complete agreement with the results observed using amplicons of regular sizes, in gDNA extracted from capillary blood. For serum separation, an ultra-low-cost, portable and hand-powered paper centrifuge was used. This paper centrifuge achieves high speeds and could be used for serum separation using only human power [14]. However, we needed to adapt it for 200 μL tubes.

The serum separated from capillary blood by this paper centrifuge could be directly used as the template in qPCR, simplifying the pre-analytic phase and eliminating the extraction steps. These shortcuts in the process could further molecular point-of-care diagnostics (e.g., in acute coronary syndromes therapy, identify carriers of the ATB-binding cassette ABCB1 3435, CYP2C19*2 and CYPC2C19*17 alleles and adjust the pharmacological approach accordingly [13]), especially in resource-poor settings. Moreover, it opens up opportunities for applications in science education and field ecology.

However, it is important to highlight that the proposed paper centrifuge has concerns regarding personal safety and infectious disease exposure during its operation (e.g., flying tubes). To outweigh these concerns, it is recommended to use personal protective equipment during the centrifugation (e.g., laboratory coats and eye protection) and seal the tubes to be centrifuged with a plastic paraffin film (e.g., Parafilm^®^ M, Bemis Company, Neenah, WI, USA).

Moreover, reducing the amplicon sizes to as small as possible (e.g., ~50 bp) to detect the highly degraded gDNA present in the serum, places some constraints on assay design: (a) The lack of space in the amplicon to include a hydrolysis probe or (b) the difficulty in discrimination between the specific PCR product from primer-dimers on melting curves when DNA intercalating dyes are used. Thus, additional PCR assay design strategies must be used to surpass these constraints: (a) A small increase in the amplicon size to accommodate a hydrolysis probe can be applied (e.g., Figure 1, 65 bp amplicon) and (b) the use of well-designed primers that do not tend to form primer-dimers, along with no template controls in the reaction and appropriate assay validation (e.g., comparison to assays using amplicon of regular sizes).

## 5. Conclusions

(a) Small DNA fragments (<100 bp) are more prevalent in serum compared to DNA of higher fragment sizes and the amount of genomic DNA enriches in the serum if the specimen remains in contact with the blood clot at room temperature; (b) Serum (without any treatment) can be used directly as the template in qPCR, exempting the requirement for DNA extraction; (c) Clinically relevant SNPs can be genotyped directly from crude serum isolated from capillary blood using a hand-powered paper centrifuge, however, amplicon of small sizes must be used.

## Figures and Tables

**Table 1:** Primer and probe sets used in serum DNA enrichment experiments.

Amplicon	Primer/Probe Sequence 5′-3′	Target
65 bp	GAGCGGCTGTCTCCACAAGT	
	HEX/TTCTGACCT/ZEN/GAAGGCTCTGCGCG/IABKFQ	*RNase P* gene
	AGATTTGGACCTGCGAGCG	
100 bp	GTAGTTTTACTTACTCTCGTCTCCACATAA	
	HEX/TGAGCAAGC/ZEN/TTTCTCACAAGCATTTGGTTT/3IABKFQ	*JAK2* gene
	CTTTGAAGCAGCAAGTATGA	
202 bp	CGCTTTGCTGTGACGCACTT	
	HEX/CTTGCCGGA/ZEN/CAGACAAAGCGTTTC/IABKFQ	*SBP2* gene
	GCAGGCGGACGGACTGAG	
688 bp	ATGGACCCTTGGCTGTCAGAATTA	
	HEX/AGCAGAAGG/ZEN/TAGAAGGCAAAGCCA/IABKFQ	*ALMS* gene
	AATGGTGTTTCCTCACATGGTCATC	

**Table 2:** Allele-specific primers sets used for SNP genotyping.

Variant	Primer Sequence 5′-3′	Commentary
**rs1801131**	**Method 1** (serum DNA)	
	GGG AGG AGC TGA CCA GTG AAa A	Reference AS primer
	GGG AGG AGC TGA CCA GTG AAa C	Alternative AS primer
	GGT AAA GAA CGA AGA CTT CAA AGA CA	Common primer
	**Method 2** (gDNA)	
	GAA CGA AGA CTT CAA AGA CAC TcT	Reference AS primer
	GAA CGA AGA CTT CAA AGA CAC TcG	Alternative AS primer
	CTC TTC TAC CTG AAG AGC AAG TCC	Common primer
	**Method 3** (gDNA)	
	CAG CAT CAC TCA CTT TGT GAC CAT T	Common primer
	Used together with method 1 specific primers	
**rs4939827**	**Method 1** (serum DNA)	
	TCA CAG CCT CAT CCA AAA GAG GAA tT	Reference AS primer
	TCA CAG CCT CAT CCA AAA GAG GAA tC	Alternative AS primer
	AGTCTGAGGGAGCTCTGGGGT	Common primer
	**Method 2** (gDNA)	
	TGA GGG AGC TCT GGG GTC CaA	Reference AS primer
	TGA GGG AGC TCT GGG GTC CaG	Alternative AS primer
	CCA GTG CCA ATC CAT CCC ATC TAT TC	Common primer
	**Method 3** (gDNA)	
	GTT TCC TCC ATG AGG AAC TCA CTC TAA AC	Common primer
	Used together with method 1 specific primers	
**rs4779584**	**Method 1** (serum DNA)	
	TCC TGT GTG TAT AGT TAT GGT TTC TGT TgG	Reference AS primer
	TCC TGT GTG TAT AGT TAT GGT TTC TGT TgA	Alternative AS primer
	CAG TAG AAC TTG TTG ATA AGC CAT TCT TC	Common primer
	**Method 2** (gDNA)	
	CAG TAG AAC TTG TTG ATA AGC CAT TCT TtC	Reference AS primer
	CAG TAG AAC TTG TTG ATA AGC CAT TCT TtT	Alternative AS primer
	GAT GAG TCC TAA CAA GGA AGG TGAC	Common primer
	**Method 3** (gDNA)	Common primer
	GAG CTG CTA TAA GAT GGG CTG AGT T	
	Used together with method 1 specific primers	
**rs3802842**	**Method 1** (serum DNA)	
	CCC TAA AAT GAG GTG AAT TTC TGG GtG	Reference AS primer
	CCC TAA AAT GAG GTG AAT TTC TGG GtT	Alternative AS primer
	CCC TTG CAG ACC CAT AGA AAA TCT	Common primer
	**Method 2** (gDNA)	
	CCC TTG CAG ACC CAT AGA AAA TCc C	Reference AS primer
	CCC TTG CAG ACC CAT AGA AAA TCc A	Alternative AS primer
	AGG ATG TTC CAC ACA GAT GCT ATC C	Common primer
	**Method 3** (gDNA)	
	CTT CCT CTG CTG TTC CTA TGA CTT C	Common primer
	Used together with method 1 specific primers	

Lower case letters indicate the deliberate mismatch introduced to enhance allele discrimination. AS—allele-specific.

**Table 3:** SNP included in this study and the amplicon size of each assay.

Variant	Gene	Global MAF	Clinical Association	Method 1 (Serum DNA)	Method 2 (gDNA)	Method 3 (gDNA)
rs1801131	*MTHFR*	0.25	Homocystinuria	51 bp	97 bp	96 bp
rs4939827	*SMAD7*	0.35	Colorectal cancer	50 bp	213 bp	105 bp
rs4779584	*SMAD7*	0.49	Colorectal cancer	59 bp	191 bp	147 bp
rs3802842	*COLCA1*	0.28	Colorectal cancer	51 bp	86 bp	274 bp
